# Facebook’s shared articles on HPV vaccination: analysis of persuasive strategies

**DOI:** 10.1186/s12889-024-19099-0

**Published:** 2024-06-24

**Authors:** Ashley Hedrick McKenzie, Elaine Avshman, Ross Shegog, Lara S. Savas, L. Aubree Shay

**Affiliations:** 1 Department of Communication, Clemson University, Clemson, SC 29634 USA; 2 Texas A&M University School of Medicine, Bryan, TX 77807 USA; 3 UTHealth Houston School of Public Health, Houston, TX 77054 USA; 4 UTHealth Houston School of Public Health, San Antonio, TX 78229 USA

**Keywords:** HPV vaccine, Social media, Persuasive strategies, Content analysis

## Abstract

**Background:**

The current study analyzed articles shared on Facebook between 2019 and 2021 that discuss the HPV vaccine. Results address a gap in knowledge about the persuasive strategies used in HPV vaccine discourse on Facebook.

**Methods:**

Using Buzzsumo.com, we collected 138 articles, shared on Facebook between 2019 and 2021, with the highest “engagement scores,” or total number of reactions, comments, and shares. Using a content analysis methodology, three independent coders were trained in using the study codebook, achieved acceptable inter-rater reliability (Krippendorf’s alpha = 0.811), and coded each article in Atlas.ti.

**Results:**

Seventy-two articles had a positive valence toward the HPV vaccine, 48 had a negative valence, and 18 were mixed-valence or neutral. Pro-vaccine articles presented a variety of evidence types in support of benefits of HPV vaccination. Pro-vaccine articles primarily originated from national and local news sources. Anti-vaccine articles combined presentation of evidence with persuasive arguments and strategies, such as mistrust of institutions, fear appeals, ideological appeals, presenting a high number of arguments or detail, and minimizing the severity of HPV. Three sources were responsible for producing 62.5% of all anti-vaccine articles in the dataset. Mixed-valence or neutral articles mixed cancer prevention discourse with ideological appeals about protecting parental rights, and were mostly produced by local news outlets.

**Conclusion:**

The results of this study can help health communicators anticipate the types of discourses that vaccine-hesitant parents may have encountered online. Implications and suggestions for practice are discussed.

## Background

Human Papillomavirus (HPV) is the most common sexually transmitted infection in the United States [1] and is a leading cause of cervical, anal, vaginal, oropharyngeal, vulvar, and penile cancers [2]. However, uptake of a vaccine that safely and effectively prevents HPV infection [3] still remains far below the U.S. Department of Health and Human Services’ Healthy People 2030 goal of 80% of adolescents completing the recommended series. In the United States, only 62.6% of adolescents aged 13 through 17 were up to date HPV vaccination [4]. Only 77.8% of girls and 74.4% of boys between 13 and 17 years old in the U.S. have initiated the HPV vaccine series [4]. 

Because HPV vaccination is recommended at age 11 or 12 [5], parents and guardians are often the primary decision-makers regarding HPV vaccination. Parents considering vaccines for their children often turn to the internet and social media for information [6, 7]. Facebook is the most popular social media platform among parents whose children are ages 9–14 [8]. However, information that parents consume via Facebook may be susceptible to inaccuracy. Previous content analyses found that Facebook posts often amplify risks of HPV vaccination, including erroneous information about safety and increasing teen sexual activity [9, 10]. One analysis of 6,506 Facebook posts about the HPV vaccine found that 39.5% of posts included messages describing the vaccine as dangerous [9]. 

A recent systematic review concluded that exposure to anti-vaccine information online has notable influences on parents [11], including associations with HPV vaccine refusal [12], lower HPV vaccination coverage at a state level [13], and greater likelihood to share negative vaccine messages on social media [13]. On the other hand, consumption of positive online content about the HPV vaccine may be associated with positive outcomes, like positive social media conversations about the vaccine and higher levels of vaccine coverage at a state level [13, 14]. 

Considering the outcomes associated with parental consumption of online content about the HPV vaccine, more research is needed to better understand the persuasive strategies accompanying (mis)information in online public discourse about the HPV vaccine. Knowledge about the range of persuasive appeals that parents encounter online will better prepare physicians and health communicators to anticipate and address parental concerns about HPV vaccination. Specifically, a focus on Facebook is warranted, as it is the most frequently used social media platform among parents whose children are ages 9–14 [8], an age range which includes the recommended age for HPV vaccination.

Existing research about public online discourse about the HPV vaccine has noted the use of several persuasive strategies. For example, the following arguments and tactics have been documented in online anti-HPV vaccine discourse: downplaying individual susceptibility to HPV; appeals to civil liberties in opposition to vaccine mandates; using personal narratives as a form of evidence; referencing doctors or other medical authority figures as a form of evidence; appealing to mistrust of Western countries (specific to platforms in Eastern countries); and appealing to mistrust of institutions, such as government or pharmaceutical companies [15–18]. Conversely, online public discourse portraying the HPV vaccine positively may also contain persuasive strategies, such as referencing peer-reviewed publications and government health sites or telling personal narratives [18, 19]. These persuasive tactics have been documented in online platforms such as Instagram, Weibo, search engine results, and other websites and forums [15–19]. However, analyses of persuasive appeals have not yet focused on Facebook. Existing research on Facebook HPV vaccine information primarily focuses on valence (pro- or anti-vaccine) and content of misinformation [9, 10]. Therefore, the current study addresses this gap by analyzing the persuasive strategies used in HPV vaccine-relevant information shared on Facebook.

Furthermore, previous research focuses on the content of Facebook posts. While valuable, these analyses do not capture the full range of discourses about the HPV vaccine that parents may encounter on Facebook. Facebook posts can often include accompanying references to long-form articles, attached via the sharing button, that are often long-form and reference a variety of wider content. Additionally, previous analyses of Facebook posts are limited to data from 2006 to 2016 and need updating. Therefore, the purpose of the current study was to analyze articles from external webpages, shared on Facebook, that discuss the HPV vaccine.

## Methods

### Data collection

We collected and analyzed 138 articles about the HPV vaccine that were widely shared on Facebook between 2019 and 2021. We used Buzzsumo.com, a web-crawling platform that indexes social engagement data, to collect articles shared on Facebook discussing the HPV vaccine. For each year (2019–2021), we collected a sample of 50 unique articles with the highest “engagement scores.” Buzzsumo calculated “engagement scores” by adding the total number of reactions, comments, and shares for each article, collected using Facebook’s API. Prior research has used Buzzsumo to identify and collect articles from Facebook with the highest engagement scores [20, 21]. When an article met study exclusion criteria, it was replaced by the article with the next highest engagement score. Duplicate articles (*n* = 11), articles not written in English (*n* = 1), and videos with no accompanying text (*n* = 1) were excluded from data collection. Given the retrospective data collection method, some links provided by Buzzsumo were no longer functional. If a link was broken, we conducted a search engine search using the article title, author name, and/or website domain name (provided by Buzzsumo). If the search did not yield any matching results, we entered the same search criteria in the Wayback Machine, a digital archive of the world wide web that provides access to archived versions of now-deleted webpages. If neither strategy yielded a matching search result, the article was excluded from data collection (*n* = 4). Finally, in order to maintain a focus on articles that were widely engaged with by Facebook users, articles with engagement scores under 1,000 were also excluded. In 2021 only 38 articles had an engagement score over 1,000. The final dataset was comprised of 138 articles.

### Data analysis

Data were analyzed using a content analysis methodology, in which the study team developed a codebook, trained independent coders in applying the codebook to the study data, and coders independently analyzed the data [22–24]. The study team developed a codebook to describe known drivers of persuasion that were either deductively identified in existing literature, or inductively identified in an initial review of the dataset. The Elaboration Likelihood Model (ELM), a cognitive processing theory [25], was selected to guide code development because its integrative framework encompasses a wide range of research findings from persuasion research. ELM describes message elements that enhance persuasion when message consumers carefully attend to the content of persuasive messages (high elaboration), or when message consumers quickly process messages without deep thinking (low elaboration).

During high elaboration, the presentation of evidence and argument quality are key drivers of persuasion. Therefore, the codebook devoted broad sections to the use of evidence and the use of persuasive arguments or strategies. Existing literature was consulted to identify specific types of evidence and persuasive arguments or strategies that are common in online public discourse about vaccination. Common types of evidence included: narratives; statistics; simple arguments; and scientific evidence [17, 26–33]. Initial team review of the dataset also resulted in the addition of codes to describe different types of evidence, such as lawsuits/legal arguments and hyperlinks to other sources. Persuasive arguments or strategies that were deductively identified in existing research included: appeals to individual choices or freedoms; emphasizing or minimizing severity of HPV; appeals to greater good/protecting others; mistrust of institutions, such as government or big pharma; emotional appeals, including fear of vaccine risks, fear of cancer risk, or positive emotions toward vaccination; and religious values [15–17, 28–30, 34, 35]. 

ELM also outlines several message elements, known as heuristic cues, that may bolster persuasion when message consumers are engaging in low elaboration [25]. During codebook development, codes were also created to describe heuristic cues inductively identified in initial review of the dataset, such as the use of authority figures and the use of a high number of arguments presented within a message. Finally, additional prevalent topics of discourse were inductively identified during initial review of the dataset, such as emphases on cancer prevention, HPV’s sexual transmission, vaccine requirements for school, and religion. Coding groups were also created to capture the year that the article was published, source of the article, and overall valence of vaccine attitudes in the article (See Table 1 for a full description of codes.) Psychology research and theory has identified positive and negative valences as key mechanisms through which humans evaluate objects [36, 37]. We added the coding group “mixed perspectives/neutral” to account for articles that included both positive and negative evaluations of the HPV vaccine.

**Table 1:** Content codes

Code	Description
Year	Use information in the article headings to assign each document to one of the following coding groups: • 2019 • 2020 • 2021
Document source	Who wrote/produced the article? Each article must be assigned to a document source using one of the following groups: • Government organization (i.e. CDC, FDA, WHO) • Hospital/medical practice/medical association/scientific publication • Insurance Company • News outlet • Non-profit/advocacy organization • Online magazine • Personal blog • Other website (includes “pseudo-news” sites that collect articles written by non-journalists)
Valence of vaccine attitudes	Consider whether the majority of the information in the article is “pro-HPV vaccine” or “anti- HPV vaccine.” Assign a document to one of the following groups after you have read the document in its entirety: • Positive valence (“pro-vaccine”) • Negative valence (“anti-vaccine”) • Mixed perspectives/neutral If an article provides an “anti-vaccine” quote or perspective, but refutes these claims or condemns them as false— then code article as “positive valence.” If an article provides quotes from both anti- and pro-vaccine speakers, but does not weigh in on the accuracy of these claims—then code the article as “mixed perspectives.” In other words, think of documents in the mixed perspectives group as giving a podium to both sides of the argument .
Evidence: “statistics”	Statistics or numbers that are related to the vaccine or the virus.
Evidence: lawsuit/legal argument	Description of an impending lawsuit or legal perspective used either in favor or against HPV vaccination. Could include mentions of lawsuits brought against the pharmaceutical company as reasons not to trust the HPV vaccine, disapproval of mandates requiring vaccination (even if these mandates are fictional), or other legislative documents such as bills or executive orders.
Evidence: link to another source	The article includes a hyperlink to another article/post/information source to bolster their own argument/claim. The article may or may not provide much context about the hyperlinked source. Exclude links that are posted after the conclusion of an article (i.e. promoting other stories on the platform).
Evidence: scientific research	The article quotes or paraphrases findings from a scientific research study.
Evidence: scientific authority figure voice	The author of a study, an academic researcher (epidemiologist, public health PhD or MA, etc.), or professor is quoted or paraphrased AND their affiliation and title are included. If a professor or researcher is identified as “Dr.,” this person may also be coded “medical authority figure voice”
Evidence: medical authority figure voice	The article quotes or paraphrases a doctor, or other medical authority figure as a form of evidence in favor or against the HPV vaccine. Include instances when an academic researcher is identified as “Dr.,” or in a way that a general audience wouldn’t be able to distinguish them from a medical doctor. This code does not include medical associations (see public health authority figure voice).
Evidence: public health authority figure voice from a government organization	This article quotes or paraphrases a public official from a health-related government organization (CDC, WHO, FDA, state/county-level public health department, medical association, other well-recognized organizations that support research, such as the American Cancer Society or Cancer Research UK) OR the organization itself. This code can also capture times where government organizations/medical associations are “name dropped” in the text.
Evidence: political authority figure voice	The article quotes or paraphrases a politician, government official, etc. as a form of evidence in favor or against the HPV vaccine. Do not include quotes from public health government officials (i.e. CDC director, etc.).
Evidence: legal authority figure voice	The article quotes or paraphrases a judge, lawyer, attorney, or legal scholar.
Evidence: non-profit authority figure voice	The article quotes or paraphrases a spokesperson from a non-profit or charitable organization related to cervical cancer or health more broadly.
Evidence: personal narrative	The article gives a story, anecdote, personal testimony, etc. from an individual as a form of evidence to bolster their argument as to why someone should or shouldn’t get the HPV vaccine. The story is about what happened to someone. It can be an individual talking about what happened to themselves or someone else.
Persuasive tactic: fear appeal	The article makes a statement clearly intended to make readers fearful of the vaccine or the consequences of not being vaccinated. The text emphasizes the potential danger and harm that will befall individuals who do not adopt the message’s recommendations. These statements could include references to death from cervical cancer, severe and life-altering side effects of the vaccine, poor health outcomes from cancer, etc. Fear appeals could be pro- or anti-vaccine.
Persuasive tactic: minimizing severity	The article makes a statement downplaying the consequences of HPV infection, the prevalence of HPV infection, etc. This persuasive tactic could be pro- or anti-vaccine.
Persuasive tactic: mistrust of institutions	The article makes statements conveying mistrust of institutions such as “big pharma,” government, and health authorities. Examples could include questioning the motives of the pharmaceutical companies that produce HPV vaccines, questioning the accuracy of government data about vaccine safety, questioning medical expertise, questioning the accuracy of health authorities’ decisions, etc.
Persuasive tactic: number of arguments/information heuristic	The article uses a particularly high number of arguments to bolster their claim that you should/shouldn’t get the HPV vaccine. Arguments may be long-winded, difficult to follow, or jargon-filled. You may end up coding large passages of text for this code. Most likely, you will use this code for at least a paragraph-worth of text. Continue to use other codes within the paragraph.
Persuasive tactic: ideological assertion	The article uses a political argument to bolster their claim you should or shouldn’t get vaccinated. Examples include: appeals to the importance of individual freedoms, threats to parental consent, dislike of government vaccine mandates, statements associating vaccination with a political party or orientation, statements about protecting vulnerable people, statements about the greater good/good of the community. This code could refer to pro- or anti- vaccine arguments.
Other discourse: School-related discourse	The article talks about schools mandating or encouraging HPV vaccination. This code will likely overlap with others (i.e. a whole paragraph coded as “school related discourse” contains a sentence also coded as coded “persuasive tactic: ideological assertion”). Do not use this code when the text references school-aged children.
Other discourse: Religious discourse	The article includes language about religion (example, religious beliefs that sex should be saved for marriage, etc.)
Other discourse: Cancer prevention discourse	Use this code to capture text in the article that frames or emphasizes the HPV vaccine as a method of cancer prevention. This does not include statements mentioning that HPV causes cancer; prevention must be mentioned or implied. (i.e. HPV causes cancer, and the vaccine prevents HPV). Can also apply to statements saying the vaccine does not prevent cancer.
Other discourse: Sexually transmitted disease discourse	Use this code to capture text in the article that describes HPV transmission via sexual contact or skin-to-skin contact, or that otherwise emphasizes HPV as an STD or the HPV vaccine’s connection to a sexually transmitted disease. Could include references to genital warts.

Three independent coders were trained in using the codebook. After conducting preliminary coding of 5 articles during training, the coders refined codebook definitions to better reflect the data. Next, the three independent coders achieved acceptable inter-rater reliability for article-level coding on a subset of 20 articles (Krippendorf’s alpha = 0.811). After achieving reliability, the coders divided the remaining articles for independent coding in Atlas.ti Web. Results, including counts and percentages, are reported at the article-level as unit of analysis.

## Results

Facebook engagement scores for articles in the dataset ranged from 1,095 to 236,841. Out of the total 138 articles, a majority were positive in valence toward the HPV vaccine (“pro-vaccine,” 72 articles), over a third were negative in valence toward the HPV vaccine (“anti-vaccine,” 49 articles), and 18 were coded as “mixed perspectives/neutral.” Mixed perspective/neutral articles gave voice to both anti- and pro-vaccine voices or perspectives, without weighing in on the accuracy of either perspectives’ claims or without correcting misinformation, or did not include any anti- or pro-vaccine perspectives. See Table 2 for code frequencies by article valence.

**Table 2:** Description of vaccine articles by valence

Category Description parameters_a_				Articles			
		Pro- Vaccination *n* = 72		Anti- Vaccination *n* = 48		Mixed Perspective *n* = 18	
		n	%	n	%	n	%
***Document source***	News outlet	45	63	4	8	16	89
	Other website	9	13	24	50	1	6
	Medical entity	7	10	1	2	n/a	n/a
	Personal blog	6	8	3	6	n/a	n/a
	Government organization (e.g., CDC)	2	3	n/a	n/a	n/a	n/a
	Online magazine	2	3	1	2	1	6
	Nonprofit/advocacy organization	1	1	15	31	n/a	n/a
***Types of evidence***	Statistics	68	94	28	58	6	33
	Public health authority figures	63	88	27	56	13	72
	Links to other sources	57	79	43	90	9	50
	Scientific research	54	75	21	44	4	22
	Scientific authority figures	36	50	12	25	3	17
	Medical authority figures	17	24	15	31	2	11
	Personal narrative	12	17	26	54	2	11
	Political authority figures	6	8	9	19	4	22
	Non-profit authority figures	5	7	6	13	n/a	n/a
	Lawsuit/legal argument	4	6	32	67	14	78
	Legal authority figures	1	1	20	42	4	22
***Persuasive tactics***	Fear appeal	8	11	32	67	1	6
	Mistrust of institutions	4	6	36	75	2	11
	Ideological assertions	3	4	23	48	10	56
	Number of arguments	3	4	14	29	n/a	n/a
	Minimizing severity of HPV	2	3	12	25	n/a	n/a
***Other discourse topics***	Cancer prevention discourse	65	90	22	46	10	56
	Sexually transmitted disease discourse	51	71	11	23	11	61
	School-related discourse	16	22	13	27	13	72
	Religious discourse	1	1	2	4	2	11

^a^Each document was coded for multiple types of evidence, persuasive tactics, and other discourse topics. Document sources were the only category of codes that were mutually exclusive

### Article sources

#### Pro-vaccine sources

News outlets were the most popular source (*n* = 45 articles) of pro-vaccine articles, and mostly originated from national or large metropolitan news outlets. Other websites (*n* = 9) included health, parenting, or other information-focused websites. Medical entities that were document sources (*n* = 7) included medical associations, research publications, and informational websites sponsored by health practices or networks.

### Anti-vaccine sources

The most common sources for negative valence articles were “Other websites” (*n* = 24) and non-profit/advocacy organizations (*n* = 15). Some of the “other” websites were pseudo-news websites, written using typical conventions of news reporting, including “news” in the website name and naming the “journalist” or “contributor” who wrote the article. However, these websites were usually sponsored by politically-oriented non-profit organizations, or listed partisan values in the website “about” page. In addition to pseudo-news websites, other sources coded as “other websites” included information, innovation, or vaccine-branded websites. Notably, a few websites were responsible for a large portion of the anti-vaccine articles. Ten articles originated from a website titled “Vaccine Impact” and 8 articles from a website called “Collective Evolution.” Similarly, twelve out of the fifteen anti-vaccine “non-profit/advocacy” articles originated Children’s Health Defense’s website. Registered as a 501 non-profit and founded by American politician Robert F. Kennedy Jr., Children’s Health Defense funds lawsuits against pharmaceutical companies for alleged vaccine injuries. In 2022, Facebook and Instagram suspended Children’s Health Defense’s social media accounts due to their promotion of misinformation [38]. However, this suspension does not prevent individual users of Facebook and Instagram from sharing articles published on Children Health Defense’s website.

### Neutral or mixed-perspective sources

News outlets accounted for 16 articles. There was also one online magazine article and one “other website.” Most news outlets were local news stations or newspapers, rather than national or large metropolitan news outlets.

### Types of evidence

#### Evidence in pro-vaccine articles

Types of evidence most frequent in positive valence articles were: statistics (*n* = 68 articles), public health authority figures (*n* = 63), links to other sources (*n* = 57), scientific research (*n* = 54), and scientific authority figures (*n* = 36). These types of evidence were usually presented to support claims about the vaccine’s efficacy and safety, or about the risk of cancer from HPV.

Frequently cited statistics described population decreases in incidence of cervical cancer/pre-cancerous lesions after the introduction of the HPV vaccine, decreases in personal odds of developing cervical cancer or contracting HPV after vaccination, prevalence of cervical cancer and HPV infection, the extent to which HPV infections eventually become cancerous, and vaccine uptake in various populations.

The code “public health authority figures” captured references to government-based public health organizations—e.g. Centers for Disease Control and Prevention (CDC), U.S. Food and Drug Administration (FDA), World Health Organization (WHO), Public Health England, etc.—or employees and representatives from these organizations, that were cited within the article. Scientific authority figures were usually university professors, researchers or epidemiologists at cancer centers or institutes, or epidemiologists who authored research papers at government public health organizations. References to scientific research within pro-vaccine articles often described discrete studies and their results, mentioning the study authors and peer-reviewed journal where the study was published.

When articles included hyperlinks to other sources, they were often presented to bolster the credibility of claims or statements of fact presented in the article by providing a link to a credible source, such as a government organization or scientific research. For example, articles would preface a statement of fact with the phrase, “According to the CDC,” and embed a hyperlink within the phrase.

### Evidence in anti-vaccine articles

Common types of evidence presented in negative valence articles were: links to other sources (*n* = 43 articles), lawsuits/legal arguments (*n* = 32), statistics (*n* = 28), public health authority figures (*n* = 27), personal narratives (*n* = 26), scientific research (*n* = 21), legal authority figures (*n* = 20), medical authority figures (*n* = 15), and scientific authority figures (*n* = 12).

Links to other sources were prevalent throughout anti-vaccine articles. Like pro-vaccine articles, hyperlinks were sometimes used to connect claims with authority figures or sources of evidence, for example describing research findings then providing an accompanying hyperlink to the study. Other anti-vaccine articles used hyperlinks without providing context about the linked webpage, embedding hyperlinks within short phrases or individual words, such as “terrible risks,” “serious adverse events,” and “deceptive marketing.” It was beyond the scope of the current analysis to follow the hyperlinks to their destinations. However, by embedding numerous hyperlinks throughout the article, anti-vaccine articles created the appearance of ample evidence in support of claims about vaccine dangers and unethical behavior from pharmaceutical companies.

Lawsuits and legal arguments typically described individuals who were suing the pharmaceutical companies that produce the HPV vaccine, on the grounds of purported injuries or death resulting from vaccination. References to legal authority figures often accompanied descriptions of legal arguments/lawsuits. Notably, Robert F. Kennedy Jr., a prominent U.S. attorney and politician, was the most frequently mentioned attorney. Other lawsuits or legal arguments included lawsuits suing schools for vaccinating teens without parental consent and arguments against bills proposing to make HPV vaccination mandatory for public school attendance.

Statistics, public health authority figures, and medical authority figures were often referenced to bolster the validity of claims about the inefficacy of the HPV vaccine or about adverse effects from HPV vaccination, including death. For example, when claiming that the HPV vaccine contains harmful chemicals, such as L-histidine, polysorbate 80, and sodium borate, one article explained that the U.S. Food and Drug Administration banned these chemicals because of their “strong association with premature ovarian failure.” A different article named and quoted a nurse and doctor who, prompted by their concern about the adverse reactions they saw in patients, collaborated on research finding that silicones are “hidden toxic ingredients in Gardasil vaccines.” The article includes a hyperlink directing readers to their study. Several articles also referenced numbers from the United States Vaccine Adverse Event Reporting System or the World Health Organization Global Adverse Drug Reactions Database as a form of evidence to substantiate claims that the HPV vaccine causes serious health harms.

Similarly, most references to scientific authority figures and scientific research were invoked to strengthen or add credibility to the claims within an article. Scientific studies were often accompanied by bibliographical information and hyperlinks to the study. It was beyond the scope of the present study to evaluate the quality of scientific research referenced in anti-vaccine articles or the accuracy of these articles’ interpretations of scientific research. However, anti-vaccine articles claimed that the research they cited came from valid sources, naming peer-reviewed journals such as *Journal of the Royal Society for Medicine, Pediatrics*, and *Current Pharmaceutical Design*, or Merck’s own clinical trial data. Generally, scientific studies were framed as reliable sources of information proving that the HPV vaccine is not safe or effective. On the other hand, public health authority figures were sometimes described as sources of mistrust; 22 articles contained codes for both public health authority figures and mistrust of institutions (see pages 12 for more detail about “mistrust of institutions.”).

Personal narratives described stories about side-effects someone experienced after receiving the HPV vaccine. Stories ranged in length from a few sentences to multiple pages and often emphasized life-altering side-effects. Some personal narratives came from parents who blamed the HPV vaccine for their children’s death. Often, these narratives accompanied information about lawsuits filed against pharmaceutical companies for damages.

### Evidence in neutral or mixed-perspectives articles

Prevalent types of evidence presented in neutral or mixed-perspectives articles were: lawsuits/legal arguments (*n* = 14), public health authority figures (*n* = 13), links to other sources (*n* = 9), and statistics (*n* = 6).

Lawsuits/legal arguments primarily involved legislation that would require HPV vaccination for public school attendance. Most articles presented the legislation as a current controversy or source of debate within the community. A few articles announced such legislation neutrally, only describing the proposed bills.

Public health authority figures, such as the CDC, FDA, or state health departments, were also mentioned in most neutral or mixed-perspectives articles. Many of these articles referenced the CDC vaccine schedule or FDA approval of the HPV vaccine. Others credited the CDC when providing a wider range of information about HPV, HPV’s association with cancer, and the vaccine’s safety and efficacy. A few articles connected the CDC or FDA with anti-vaccine rhetoric, for example quoting CDC data about adverse events after HPV vaccination.

Links to other sources combined strategies for hyperlinking within both pro- and anti-vaccine articles. While some articles specified the destination of hyperlinks, naming scientific studies or government public health organizations, other articles did not name a destination for hyperlinked text describing vaccine side effects or safety concerns. Other hyperlinks were associated with proposed bills requiring HPV vaccination for school.

Approximately one third of the neutral or mixed perspectives articles included statistics. Numbers and statistics described decreases in HPV infection rates, efficacy of the vaccine, numbers of adverse events after vaccination reported, and incidence of cervical cancer.

### Persuasive tactics

#### Persuasive tactics in pro-vaccine articles

Persuasive tactics were not frequent among pro-vaccine articles. However, when used, persuasive tactics in pro-vaccine articles included: fear appeals (*n* = 8), mistrust of institutions (*n* = 4), and ideological assertions (*n* = 3). Fear appeals highlighted the severity of HPV-associated cancers or long-term consequences for survivors. For example, one article described oropharyngeal cancer survivors who required use of feeding tubes after treatment. Other fear appeals involved testimonies from individuals with severe or terminal cancer diagnoses, such as a woman with cervical cancer who said, “I just think that if it existed when I was a teenager, I wouldn’t be dying now and my son wouldn’t be facing a future as an orphan. And that’s the clearest message I can give.” The code “mistrust of institutions” described instances in which government entities failed to pass laws requiring vaccination for public school attendance or made other missteps in vaccine promotion efforts, such as the FDA initially only approving the vaccine for girls. Ideological assertions referenced the potential of the vaccine to reduce health disparities and to protect future generations.

### Persuasive tactics in anti-vaccine articles

Persuasive arguments and strategies that were frequently used in anti-vaccine articles include: mistrust of institutions (*n* = 36), fear appeals (*n* = 32), ideological assertions (*n* = 23), number of arguments (*n* = 14), and minimizing the severity of HPV (*n* = 12).

The code “mistrust of institutions” referred to claims about unethical behavior from government institutions, politicians, and pharmaceutical companies. Several articles made claims that the CDC or FDA turned a blind eye to the “dangers” of HPV vaccination and fast-tracked its approval, because of illicit deals with pharmaceutical companies that stand to gain profit from widespread HPV vaccination. Other articles claimed that government organizations and pharmaceutical companies deceptively portrayed the vaccine as safe and effective in advertisements and promotional materials about the vaccine. The code “mistrust of institutions” also described articles questioning the validity and ethics of pharmaceutical companies’ clinical trials.

Fear appeals often overlapped with personal narratives describing life-altering or deadly side effects of the vaccine. To illustrate, one article told the story of a girl who, after receiving Gardasil, developed a long list of health problems, including daily seizures, total vision loss, vertigo, and endometriosis. Her symptoms made it impossible to attend public school, and the article claimed that the HPV vaccine took her “from her idyllic life as a happy gifted child with a bright future into a nightmare existence of debilitating agony.” Of the 32 documents containing fear appeals, 23 also contained personal narratives. Fear appeals unattached to personal narratives broadly referenced the “dangerous” nature of the vaccine.

A few ideological assertions were also common throughout the negative valence articles. Bills proposing requirement of HPV vaccination for school attendance were generally discussed as government overreach violating individual choice and stripping parents of their rights regarding their children. Other articles invoked the protection of children as the basis for ideological arguments, claiming that children who have been harmed by vaccines deserve justice from big pharma’s greed. Finally, several articles invoked ideals about freedom of speech and the importance of open debate. These articles claimed that “mainstream” media’s silence about HPV vaccine dangers is suspicious, and anyone who is skeptical or nervous about vaccines often faces ridicule.

The code “number of arguments/information heuristic” described articles containing an overwhelming amount of information, in terms of number of arguments, level of detail, and/or use of jargon. This code often flagged sections of 2 or more pages worth of text, using medical jargon and detailing topics that are difficult to understand, such as the biological mechanisms through which aluminum is expelled from the body or critiques of the experimental designs used to evaluate the HPV vaccine’s safety and efficacy. The Elaboration Likelihood Model (ELM) posits that a high number of arguments within a message, or otherwise a high level of detail, might function as a heuristic cue supporting information credibility among viewers who are not highly focused on the message’s content [25]. In other words, readers who are skimming the article may perceive such in-depth content as evidence that the article is well-researched or highly credible.

Finally, anti-vaccine articles utilized the persuasive tactic of minimizing the severity of HPV. Articles that minimized the severity of HPV made claims such as: HPV usually goes away on its own, most HPV infections do not lead to cancer, few people die from cervical cancer every year, current strategies for detecting and treating cervical cancer are effective, sexually inactive children have a low risk for contracting HPV, and the risks of adverse effects from vaccination outweigh HPV-associated risks. These claims were often supported with statistics. For example, several articles repeated the claim that the chances of getting an autoimmune disease from the HPV vaccine are 1,000 times higher than the risk of dying from cervical cancer.

### Persuasive tactics in neutral or mixed-perspectives articles

Ideological assertions (*n* = 10) were the most prevalent persuasive tactic in neutral or mixed-perspectives articles. Most ideological assertions involved quotes from individuals who felt that bills requiring HPV vaccination for public school attendance infringe on parental rights. Two articles included appeals to mistrust of institutions, and one article used a fear appeal.

### Other discourse topics

#### Other discourse topics in pro-vaccine articles

“Cancer prevention discourse” (*n* = 65) was the most frequent topic of discourse in pro-vaccine articles, followed by sexually transmitted disease discourse (*n* = 51). Cancer prevention discourse included topics such as descriptions of the HPV vaccine as effective in preventing a range of cancers, statistics about decreases in cervical cancer and pre-cancers within study samples, cervical cancer incidence, and the potential for high vaccine uptake to eliminate cervical cancer at the population level. By highlighting an important benefit of vaccination, cancer prevention discourse in pro-vaccine articles often resembled “gain framing,” a persuasive strategy in which a message highlights the benefits associated with adoption of a recommended behavior [39]. 

Sexually transmitted disease discourse highlighted the fact that HPV is transmitted by sexual contact. Some articles coupled information about the sexual transmission of HPV with text explaining the importance of vaccination at a young age, long before sexual initiation. However, other articles did not make this connection clear.

### Other discourse topics in anti-vaccine articles

In anti-vaccine articles, the code “cancer prevention discourse” (*n* = 22) primarily described text questioning the effectiveness of the vaccine in preventing cancer. Anti-vaccine articles made the following claims: populations with high vaccination rates have seen increases in cervical cancer incidence; the HPV vaccine increases risk of precancerous lesions in girls and women who had already been exposed to HPV at the time of vaccination; and no one actually knows if the vaccine prevents cancer because vaccine clinical trials did not evaluate cancer incidence as an outcome. Other articles called the efficacy of the vaccine into question more subtly by using language suggesting the author’s doubt, such as saying that the vaccine was “promoted” or “marketed” by pharmaceutical companies as a way to prevent cancer.

Discourse about schools (*n* = 13) and about HPV as a sexually transmitted disease (*n* = 11) were also present in anti-vaccine articles. Focuses of school-related discourse included lawsuits filed by parents whose children were vaccinated without parental consent and disapproval of proposed legislation that would require vaccination for public school attendance. Proposed legislation was discussed as a violation of parental rights and as an unethical attempt by pharmaceutical companies to generate more profit. Discourse about the sexual transmission of HPV sometimes overlapped with discourse about schools; some articles made the argument that it is inappropriate to force children to receive a vaccine for a sexually transmitted disease.

### Other discourse topics in neutral or mixed-perspectives articles

School-related discourse (*n* = 13) in neutral or mixed-perspectives articles pertained to proposed legislation that would require HPV vaccination for public school attendance. Discourses about HPV as a sexually transmitted disease (*n* = 11) and about cancer prevention (*n* = 10) were also prevalent in neutral or mixed-perspectives articles. Although many articles merely referenced the fact that HPV is a sexually transmitted disease, others explained the need to vaccinate children before sexual initiation, or argued that the sexually transmitted nature of HPV makes the vaccine inappropriate for children. Some articles including cancer prevention discourse only briefly referenced the vaccine’s purpose to prevent cancer. Others included more in-depth content about the range of cancers prevented by vaccination, sharing statistics about the vaccine’s efficacy and explaining its potential to eliminate cervical cancer at a population level.

## Discussion

This study analyzed the persuasive strategies used in 138 of the most popular articles about the HPV vaccine that were shared on Facebook between 2019 and 2021. Seventy-two articles portrayed the vaccine in a positive light. These articles were produced by mostly national and large metropolitan news outlets and primarily relied on credible sources of information, including public health authority figures, scientific research, and scientific authority figures. They also often included statistics and hyperlinks directing readers to credible sources. The primary topic of discourse involved cancer prevention, which typically invoked a “gain” frame by describing cancer prevention as a key benefit of HPV vaccination. When describing the vaccine and HPV, positive-valence articles also often included information about sexual transmission of HPV. However, some articles discussing sexual transmission of HPV did not clearly explain the rationale for vaccinating children. This presents a missed opportunity as multiple studies have shown that framing HPV vaccination as cancer prevention, rather than discussing sexual transmission or symptoms, leads to greater vaccine acceptance [40, 41]. However, beyond presenting credible evidence within gain framing, pro-vaccine articles rarely utilized additional persuasive arguments or tactics. This finding indicates a missed opportunity for news media and other health communicators to effectively promote HPV vaccination. Fear appeals, describing personal stories from individuals who have suffered from or died from HPV-associated cancers, were used in a small number of pro-vaccine articles and may be especially effective in enhancing persuasion when paired messages about the efficacy of the vaccine [42, 43]. 

Out of the 48 articles with a negative valence toward the HPV vaccine, most originated from an “other website,” including pseudo-news websites, or websites of non-profit organizations created with the goal of advocating against HPV vaccination. A small number of websites and non-profits were responsible for the bulk of this content; three sources produced 30 of the articles (62.5% of the anti-vaccine articles). Existing research reports similar findings about a small number of sources producing a large amount of widely-spread misinformation on social media [44, 45]. Facebook, Instagram, and Twitter have tried to combat the spread of misinformation by inactivating online accounts known for spreading misinformation [38]. However, our results show that misinformation spreaders can still proliferate on social media via sharing functions, through which social media users can share articles from outside websites. Alternative strategies utilized by social media platforms, such as tags or warning labels on posts sharing misinformation, may be ineffective in mitigating the impact of misinformation on viewers’ beliefs [46]. Future research should continue to explore strategies for targeting the spread of misinformation originating from a small number of unmoderated websites, that is then shared on social media platforms.

Articles portraying the HPV vaccine in a negative light differed from pro-vaccine articles in their presentation of evidence and use of additional persuasive strategies. Lawsuits, legal arguments, and legal authority figures were presented as forms of evidence of dangerous side effects from the HPV vaccine. Personal narratives often accompanied information about lawsuits, describing harrowing stories about those who were purportedly harmed or killed by the vaccine. Other forms of evidence, including scientific research, scientific authority figures, medical authority figures, and statistics, were also presented as reliable sources of information about the harms or inefficacy of the HPV vaccine. Out of the forms of evidence coded for, only public health authority figures were, at times, discussed as a source of mistrust. Still, many anti-vaccine articles referenced information or data from sources like the CDC or FDA in support of anti-vaccine arguments or claims. Also, anti-vaccine articles strategically used hyperlinks to create the appearance of ample evidence in support of vaccine harms or inefficacy, embedding hyperlinks throughout many of the article’s claims without providing context about the hyperlinks’ destinations. Cancer prevention discourse within these articles repeated misinformation claims that the HPV vaccine causes cervical cancer, rather than preventing it. Discourse about the sexual transmission of HPV emphasized the idea that it is inappropriate to vaccinate children for a sexually transmitted disease. Anti-vaccine articles included an additional topic of discussion, focused on disapproval or outrage for proposed legislation that would require HPV vaccination for public school attendance.

Furthermore, unlike pro-vaccine articles, articles with a negative valence toward the HPV vaccine used a variety of persuasive tactics and arguments, beyond presenting evidence. Mistrust of institutions, especially regarding pharmaceutical companies and government institutions, was coded in 75% of anti-vaccine articles. Fear appeals emphasized the severity of dangers attributed to the vaccines, often highlighting personal narratives from people who believe they were harmed, or that their child died, as a result of vaccination. Ideological appeals in anti-vaccine articles referenced ideals of protecting children, protecting parental rights, and protecting freedom of speech. Some articles minimized the severity of HPV by downplaying the risk of developing cancer from HPV and claiming that the HPV vaccine’s harms outweigh any potential benefit. Finally, some anti-vaccine articles presented an overload of information, in terms of number of arguments, detail of information, and use of jargon. The Elaboration Likelihood Model suggests that these passages, sometimes spanning multiple pages of text, may act as a “heuristic cue” or mental shortcut for anyone not reading the message closely—at a glance conveying the sense that the article is written by someone with in-depth knowledge and enhancing the perceived credibility of the source [25]. Similar persuasive strategies have been identified in social media discourse about the COVID-19 vaccine, such as concerns about efficacy, safety, and side effects; mistrust of government and health institutions; and minimizing severity of COVID-19 infection [47]. Given the sustained prevalence of these themes in social media discourse about different vaccines, future health promotion efforts for vaccines might consider pre-emptively addressing these topics; inoculation theory posits that exposure to a weakened version of misinformation, accompanied with debunking information that gives advice on how to spot and refute misinformation, in effect “immunizes” against misinformation in the future [48]. 

The dataset also included 18 mixed-valence or neutral articles about the HPV vaccine. Most of these articles were produced by local news outlets. The prevalence of local news within mixed-valence articles aligns with research finding that local journalists highly value the goal of providing the community with a forum for public discourse [49, 50]. It seems likely that local journalists, who perceive creating a community forum as an important function of local news, would be more likely to seek out and report “both sides” of the debate about HPV vaccination. Mixed-valence/neutral articles primarily focused on proposed legislation that, if passed, would require HPV vaccination for school attendance. Public health authority figures and statistics were referenced among both pro and anti-vaccine perspectives, and ideological assertions touted the protection of parental rights against government infringement. Mixed-valence articles described the HPV vaccine as a method of cancer prevention and described the sexual transmission of HPV, sometimes making the argument that it is inappropriate to give children a vaccine for a sexually transmitted disease.

The current study has several limitations. While this study describes the content of articles that were highly shared on Facebook, our results do not directly speak to parents’ attitudes, beliefs, and vaccine intentions. Additionally, the current study did not include data about the demographic information of people who shared and interacted with the articles; it is possible that Facebook users without vaccine-eligible children drove engagement with these articles. Finally, data collection was limited to articles posted between 2019 and 2021. This time frame was determined by the limitations of Buzzsumos’ retrospective data-collection abilities, which allow users to collect two years of historical data beyond the current year. Discourse about the HPV vaccine may have differed before 2019, and may have changed after 2021. Notably, these years spanned the COVID-19 pandemic. Initial team review of the dataset found few direct references to the pandemic, so COVID-19 discourse was not included in the codebook for the present study. However, in a separate analysis we analyzed changes in HPV-related content across the three years and found that both content and valence differed by year.

Still, pediatricians and health communicators can use the results from this study to anticipate the concerns or perspectives that vaccine-hesitant parents may have encountered online. Given the prevalence of ideological appeals warning about suppression of questions or concerns about the HPV vaccine in the dataset, it may be prudent to thoughtfully hear out and address hesitant parents’ concerns and questions. Additionally, pediatricians and health communicators should be prepared to address concerns about lawsuits claiming vaccine damages and about the reliability of existing evidence about the vaccine’s safety. Pharmaceutical companies and government-associated public health organizations were sometimes treated with suspicion, but anti-vaccine articles usually presented evidence from scientific research, scientific authority figures, and medical authority figures as trustworthy. It may be useful to highlight these information sources in patient-provider conversations and in educational literature, rather than CDC guidelines or pharmaceutical companies’ clinical trials. Finally, the lack of persuasive tactics and arguments present in pro-vaccine articles highlights a missed opportunity that health communicators should address by integrating persuasive appeals into HPV vaccine messaging.

## Conclusion

The results of this study add to the current scope of knowledge about HPV vaccine misinformation by documenting the types of evidence presented and persuasive tactics used in long-form articles shared on Facebook. Some of the prevalent themes in the current analysis align with findings from existing research about online HPV vaccine discourse, including: arguments minimizing severity of HPV; appeals to civil liberties in opposition to vaccine mandates; appealing to mistrust of institutions, such as government or pharmaceutical companies; and using a variety of forms of evidence, such as doctors, scientific research, government health authority figures, and personal narratives forms of evidence [15–19]. However, the current study also detected additional themes unidentified in previous research, such as the use of lawsuits as a form of evidence of the HPV vaccine’s dangerous side effects, presenting unwieldy amounts of information as a potential persuasive strategy, and gratuitous use of hyperlinks to create the appearance of ample evidence.

Patient or Public Contribution: The current study involves in-depth analysis of health misinformation. Exposure to health misinformation may be associated with harmful health outcomes. Therefore, it would be unethical to expose patients or members of the public to false health information, and so the current study does not involve patients or members of the public in the study design.

## Data Availability

The datasets used and/or analyzed during the current study are available from the corresponding author on reasonable request.

## Abbreviations

HPV: Human papillomavirus
ELM: Elaboration Likelihood Model
CDC: Centers for Disease Control and Prevention
FDA: U.S. Food and Drug Administration
WHO: World Health Organization

## References

[1] Kreisel KM, Spicknall IH, Gargano JW, Lewis FM, Lewis RM, Markowitz LE (2021). Sexually transmitted infections among US women and men: prevalence and incidence estimates, 2018. Sex Transm Dis.

[2] Viens LJ, Henley SJ, Watson M, Markowitz LE, Thomas CC, D.Thompson T (2016). Human papillomavirus–associated cancers — United States, 2008–2012. Morb Mortal Wkly Rep.

[3] Meites E, Kempe A, Markowitz LE (2016). Use of a 2-dose schedule for human papillomavirus vaccination — updated recommendations of the Advisory Committee on Immunization practices. Morb Mortal Wkly Rep.

[4] Pingali C. Vaccination Coverage Among Adolescents Aged 13–17 Years — National Immunization Survey–Teen, United States, 2022. MMWR Morb Mortal Wkly Rep [Internet]. 2023 [cited 2023 Dec 21];72. https://www.cdc.gov/mmwr/volumes/72/wr/mm7234a3.htm. 10.15585/mmwr.mm7234a3 PMC10468222 37616185

[5] Centers for Disease Control and Prevention. HPV Vaccination: For Providers [Internet]. 2021 [cited 2023 Dec 21]. https://www.cdc.gov/vaccines/vpd/hpv/hcp/index.html.

[6] Ashfield S, Donelle L (2020). Parental Online Information Access and Childhood Vaccination Decisions in North America: scoping review. J Med Internet Res.

[7] Melovic B, Jaksic Stojanovic A, Vulic TB, Dudic B, Benova E (2020). The impact of online media on parents’ attitudes toward Vaccination of Children—Social Marketing and Public Health. Int J Environ Res Public Health.

[8] Manganello JA, Chiang SC, Cowlin H, Kearney MD, Massey PM (2023). HPV and COVID-19 vaccines: social media use, confidence, and intentions among parents living in different community types in the United States. J Behav Med.

[9] Luisi MLR (2021). From bad to worse II: risk amplification of the HPV vaccine on Facebook. Vaccine.

[10] Sundstrom B, Aylor E, Cartmell KB, Brandt HM, Bryant DC, Hughes Halbert C (2018). Beyond the birds and the bees: a qualitative content analysis of online HPV vaccination communication. J Communication Healthc.

[11] Ortiz RR, Smith A, Coyne-Beasley T (2019). A systematic literature review to examine the potential for social media to impact HPV vaccine uptake and awareness, knowledge, and attitudes about HPV and HPV vaccination. Hum Vaccines Immunotherapeutics.

[12] Margolis MA, Brewer NT, Shah PD, Calo WA, Gilkey MB (2019). Stories about HPV vaccine in social media, traditional media, and conversations. Prev Med.

[13] Dunn AG, Surian D, Leask J, Dey A, Mandl KD, Coiera E (2017). Mapping information exposure on social media to explain differences in HPV vaccine coverage in the United States. Vaccine.

[14] Britt RK, Hatten KN, Chappuis SO (2014). Perceived behavioral control, intention to get vaccinated, and usage of online information about the human papillomavirus vaccine. Health Psychol Behav Med.

[15] Chen L, Ling Q, Cao T, Han K (2020). Mislabeled, fragmented, and conspiracy-driven: a content analysis of the social media discourse about the HPV vaccine in China. Asian J Communication.

[16] Madden K, Nan X, Briones R, Waks L (2012). Sorting through search results: a content analysis of HPV vaccine information online. Vaccine.

[17] Massey PM, Kearney MD, Hauer MK, Selvan P, Koku E, Leader AE (2020). Dimensions of Misinformation about the HPV vaccine on Instagram: Content and Network Analysis of Social Media Characteristics. J Med Internet Res.

[18] Penţa MA, Băban A (2014). Dangerous Agent or saviour? HPV Vaccine representations on Online discussion forums in Romania. IntJ Behav Med.

[19] Keelan J, Pavri V, Balakrishnan R, Wilson K (2010). An analysis of the human papilloma virus vaccine debate on MySpace blogs. Vaccine.

[20] Allcott H, Gentzkow M, Yu C (2019). Trends in the diffusion of misinformation on social media. Res Politics.

[21] Waszak PM, Kasprzycka-Waszak W, Kubanek A (2018). The spread of medical fake news in social media – the pilot quantitative study. Health Policy Technol.

[22] Riffe D, Lacy S, Fico F, Watson B (2019). Analyzing media messages: using quantitative content analysis in Research.

[23] Sell TK, Hosangadi D, Trotochaud M (2020). Misinformation and the US Ebola communication crisis: analyzing the veracity and content of social media messages related to a fear-inducing infectious disease outbreak. BMC Public Health.

[24] Ramanadhan S, Mendez SR, Rao M, Viswanath K (2013). Social media use by community-based organizations conducting health promotion: a content analysis. BMC Public Health.

[25] Petty R, Briñol P. The Elaboration Likelihood Model. Handbook of theories of social psychology: Collection: volumes 1 & 2. SAGE; 2011. pp. 224–45.

[26] Boatman DD, Eason S, Conn ME, Kennedy-Rea SK (2022). Human papillomavirus vaccine messaging on TikTok: Social Media Content Analysis. Health Promot Pract.

[27] Broniatowski DA, Hilyard KM, Dredze M (2016). Effective vaccine communication during the disneyland measles outbreak. Vaccine.

[28] Greenberg J, Dubé E, Driedger M (2017). Vaccine hesitancy: in search of the Risk Communication Comfort Zone. PLoS Curr.

[29] Ihlen Ø, Toledano M, Just SN. Using Rhetorical Situations to Examine and Improve Vaccination Communication. Frontiers in Communication [Internet]. 2021 [cited 2023 Dec 21];6. https://www.frontiersin.org/articles/10.3389/fcomm.2021.697383.

[30] Lawrence HY (2018). When patients question vaccines: considering Vaccine Communication through a Material Rhetorical Approach. Rhetoric Health Med.

[31] Moran MB, Lucas M, Everhart K, Morgan A, Prickett E (2016). What makes anti-vaccine websites persuasive? A content analysis of techniques used by anti-vaccine websites to engender anti-vaccine sentiment. J Communication Healthc.

[32] Okuhara T, Ishikawa H, Okada M, Kato M, Kiuchi T. Persuasiveness of Statistics and Patients’ and Mothers’ Narratives in Human Papillomavirus Vaccine Recommendation Messages: A Randomized Controlled Study in Japan. Frontiers in Public Health [Internet]. 2018 [cited 2023 Dec 21];6. https://www.frontiersin.org/articles/10.3389/fpubh.2018.00105. 10.3389/fpubh.2018.00105 PMC5906532 29707533

[33] Xu Y, Margolin D, Niederdeppe J (2021). Testing strategies to increase source credibility through Strategic Message Design in the Context of Vaccination and Vaccine Hesitancy. Health Commun.

[34] Lee MSW, Male M. Against medical advice: the anti-consumption of vaccines. Fortin D, Uncles M, editors. Journal of Consumer Marketing. 2011;28(7):484–90.

[35] Li J, Zheng H (2020). Coverage of HPV-Related information on Chinese Social Media: a content analysis of Articles in Zhihu. Hum Vaccines Immunotherapeutics.

[36] Lewin K (1942). Field theory and learning. The forty-first yearbook of the National Society for the Study of Education: part II, the psychology of learning.

[37] Valence. (2018). In: *APA Dictionary of Psychology*.

[38] Klepper D. RFK Jr.’s anti-vaccine group kicked off Instagram, Facebook. Associated Press News [Internet]. 2022 Aug 18 [cited 2023 Dec 21]; https://apnews.com/article/covid-technology-health-public-misinformation-28019177323c1d50b7ff28c522dde083.

[39] Rothman AJ, Salovey P (1997). Shaping perceptions to motivate healthy behavior: the role of message framing. Psychol Bull.

[40] Leader AE, Weiner JL, Kelly BJ, Hornik RC, Cappella JN (2009). Effects of Information Framing on Human Papillomavirus Vaccination. J Women’s Health.

[41] McRee AL, Reiter PL, Chantala K, Brewer NT (2010). Does Framing Human Papillomavirus Vaccine as preventing Cancer in men increase vaccine acceptability? Cancer Epidemiology. Biomarkers Prev.

[42] Carcioppolo N, Jensen JD, Wilson SR, Collins WB, Carrion M, Linnemeier G (2013). Examining HPV threat-to-efficacy ratios in the extended parallel process model. Health Commun.

[43] Reno JE, Dempsey AF (2023). Promoting HPV vaccination among Latinx: an application of the extended parallel processing model. J Behav Med.

[44] Ceylan G, Anderson IA, Wood W. Sharing of misinformation is habitual, not just lazy or biased. Proceedings of the National Academy of Sciences. 2023;120(4):e2216614120. 10.1073/pnas.2216614120 PMC9942822 36649414

[45] Nogara G, Vishnuprasad PS, Cardoso F, Ayoub O, Giordano S, Luceri L. The Disinformation Dozen: An Exploratory Analysis of Covid-19 Disinformation Proliferation on Twitter. In: Proceedings of the 14th ACM Web Science Conference 2022 [Internet]. New York, NY, USA: Association for Computing Machinery; 2022 [cited 2023 Dec 21]. pp. 348–58. (WebSci ’22). 10.1145/3501247.3531573.

[46] Sharevski F, Alsaadi R, Jachim P, Pieroni E (2022). Misinformation warnings: Twitter’s soft moderation effects on COVID-19 vaccine belief echoes. Computers Secur.

[47] Cascini F, Pantovic A, Al-Ajlouni YA, Failla G, Puleo V, Melnyk A et al. Social media and attitudes towards a COVID-19 vaccination: A systematic review of the literature. eClinicalMedicine [Internet]. 2022 Jun 1 [cited 2023 Dec 21];48. https://www.thelancet.com/journals/eclinm/article/PIIS2589-5370(22)00184-5/fulltext. 10.1016/j.eclinm.2022.101454 PMC9120591 35611343

[48] Traberg CS, Roozenbeek J, van der Linden S (2022). Psychological inoculation against misinformation: current evidence and future directions. ANNALS Am Acad Political Social Sci.

[49] Franklin B. Local journalism and local media: making the Local News. Routledge; 2006. p. 359.

[50] Hanusch FA, Different Breed (2015). Altogether? Journalism Stud.

